# Genome Sequencing of *Mesonia algae* K4-1 Reveals Its Adaptation to the Arctic Ocean

**DOI:** 10.3389/fmicb.2019.02812

**Published:** 2019-12-04

**Authors:** Ran Huan, JiaFeng Huang, Dan Liu, Meng Wang, CongLing Liu, YunQian Zhang, CuiPing Yi, Dong Xiao, HaiLun He

**Affiliations:** ^1^School of Life Sciences, Central South University, Changsha, China; ^2^School of Chemistry and Biological Engineering, Changsha University of Science and Technology, Changsha, China; ^3^State Key Laboratory of Coal Resources and Safe Mining, China University of Mining and Technology, Xuzhou, China

**Keywords:** *Mesonia*, complete genome, environmental adaptation, the Arctic Ocean, salt tolerance

## Abstract

The special ecological environment of the Arctic has brought about a large number of salt-tolerant and psychrotolerant microorganisms. We isolated two culturable bacterial strains of the genus *Mesonia*; one from the Arctic ocean, *Mesonia algae* K4-1, and one from the tropical sea, *Mesonia* sp. HuA40. Our genome analysis and phenotypic experiments indicated that *Mesonia algae* K4-1 is a moderately halophilic and psychrophilic bacterium. *Mesonia algae* K4-1 can tolerate 3–14% NaCl and grow at a wide range of temperatures from 4 to 50°C. *Mesonia* sp. HuA40 is a mesophilic bacterium that can only grow with 3–9% NaCl. In addition, the salt adaptation strategy of *Mesonia algae* K4-1 accumulates organic osmolytes in the cell. RNA helicases, glutathione and organic compatible solutes may play important roles in maintaining the metabolism and physiological function of *Mesonia algae* K4-1 under cold stress. Moreover, the ability of *Mesonia algae* K4-1 to adapt to an oligotrophic marine environment is likely due to the synthesis of a large number of extracellular polysaccharides and the secretion of various families of extracellular proteases. This study systematically analyzed the relationship between genomic differentiation and environmental factors of the *Mesonia* genus and revealed the possible adaptation mechanism of *Mesonia algae* K4-1 in the extreme Arctic marine environment at the genomic level.

## Introduction

The polar regions constitute up to 14% of the cold habitats on Earth. The Arctic is very cold and harsh for most of the year with little solar radiation. One of the unique characteristics of Arctic is the periodical melting of sea ice. Bacteria in the Arctic may face a sudden drop in ambient salt concentration when the ice melts (Thomas and Dieckmann, 2002). The capacity for polar microbes to exist and proliferate in this extreme environment means that they have overcome key intrinsic obstacles to survive in cold environments (D’Amico et al., 2006). Many halophilic and psychrophilic bacteria have been isolated from the Arctic ocean (Van Trappen et al., 2004; Yukimura et al., 2010). However, how these microbes are adapted to survive environmental changes in extreme conditions is largely unknown.

Different species of the same genus from different ecosystems may evolve different biochemical and physiological properties (Konstantinidis et al., 2009; Chen et al., 2010; Qin et al., 2010, 2014). Comparative genomics can offer detailed descriptions of bacterial growth, evolution, and phylogeny (Daubin et al., 2003; Descorps-Declere et al., 2008; Caputo et al., 2019). It has become a powerful method to exploit the relationship between environmental adaption and species evolution. Pittera et al. (2018) revealed a specific adaptability to the content of lipid desaturase by conducting comparative genomic studies on membrane lipid biosynthesis pathways of 53 marine *Synechococcus* strains from different ecotypes. Omae et al. (2017) used comparative genomic analysis to show the adaptability of *C. maritimus* KKC1 to submerged marine caldera; *C. maritimus* KKC1 lacks specific electron-bifurcating enzymes and possesses six CO dehydrogenases.

The genus *Mesonia*, belonging to the family Flavobacteriaceae within the phylum Bacteroidetes, was first discovered by Nedashkovskaya et al. (2003). Currently, a total of 8 species of *Mesonia* have been validly described (Nedashkovskaya et al., 2003, 2006; Kang and Lee, 2010; Lee et al., 2012; Choi et al., 2015; Kolberg et al., 2015; Wang et al., 2015; Sung et al., 2017). All species isolated from the ocean and some additional species can hydrolyze gelatin (Nedashkovskaya et al., 2003; Sung et al., 2017), casein (Nedashkovskaya et al., 2003; Choi et al., 2015; Sung et al., 2017), fucoidan (Urvantseva et al., 2006), Tween 80 (Nedashkovskaya et al., 2006; Kang and Lee, 2010; Choi et al., 2015; Wang et al., 2015). Earlier studies found that some strains of the genus can form biofilms (Jeong et al., 2016) and are multiresistant (Miranda et al., 2015). Biofilms afford bacterial stability in the growing environment, allow for the capture of nutrients, and provide a range of environmental challenges and pressure protection (Ricciardelli et al., 2019). However, the current research on this genus is still very limited, and most of the existing reports are about their uncultivable research. No genome of the genus *Mesonia* isolated from the Arctic has been sequenced. Therefore, a genome and physiological characteristic study of the genus *Mesonia* is beneficial for exploring its potential and development as a type of biological resource.

In this work, we isolated two strains of the genus *Mesonia* from different niches and found differences in their physiological and biochemical characteristics. *Mesonia algae* K4-1 is a typical psychrophilic and moderate halophilic bacterium isolated from the Arctic (12°07.553E, 78°55.464 N). *Mesonia* sp. HuA40 was isolated from hull attachments at the bottom of a ship located in the tropical marine Zhanjiang, Guangdong Province, China (110°96.191E, 20°88.073N). Strain K4-1 is an ideal material for studying how the Arctic bacteria of the genus *Mesonia* are adapted to survive in extreme conditions. Considering the species diversity and metabolic plasticity of polar bacteria, more representative sequences must be analyzed to fully describe the special characteristics. Therefore, we analyzed the genome sequence and phenotypic characterization focusing on the study of genes related to several metabolic processes that are crucial to environmental adaptability, such as salt tolerance, cold adaptation, exopolysaccharide production and degradation of proteinaceous organic nitrogen. This research provides insights into the survival mechanism of the *Mesonia* group from a genomic perspective.

## Materials and Methods

### Bacterial Strains and Genomic DNA Extraction

*Mesonia algae* K4-1 was collected at 117 m depth in the Arctic (12°07.553E, 78°55.464N). The strain *Mesonia* sp. HuA40 was isolated from hull attachments in Zhanjiang, Guangdong Province, China (110°96.191E, 20°88.073N). The two strains were cultured in 2216E medium containing 5 g of peptone, 1 g of yeast extract, and 0.01 g of Fe_2_(PO4)_3_ in 1 L of artificial seawater, pH 7.5 (Wu et al., 2016). The 16S rRNA genes were sequenced to validate the obtained strains. Genomic DNA was prepared using a genomic DNA extraction kit (Biospin, China) according to the manufacturer’s instructions. The two strains were deposited in the China Center for Type Culture Collection (CCTCC), the collection number for *Mesonia algae* K4-1 is CCTCC M 2018482, and the collection number for the *Mesonia* sp. HuA40 is CCTCC NO:M 2018481.

### Draft Genome Sequence

Whole genome sequencing of the two strains was performed using the Illumina HiSeq 3000 (Illumina, Inc., United States) at Genergy Biotechnology (Shanghai, China). Skewer software was used to dynamically remove joint sequence fragments and tail segments with a mass value lower than Q30 from the 3′ end of the sequence, erase segments with a mean value lower than Q30, and remove segments with lengths less than 50 bp. Reads were assembled into contigs using SPAdes (version: 3.5.0), and contig less than 500 bp in length were removed. Gene prediction software Prokka^1^ was used to predict the assembled sequences.

### Genome Annotation and Analysis

Putative coding sequences (CDSs) were identified by Glimmer 3.0 (Delcher et al., 2007). Subsequently, protein sequences were further analyzed on the basis of Swiss-Prot (Magrane and UniProt, 2011), KEGG (Kanehisa et al., 2008), and eggNOG databases (evalue < 0.00001) (Huerta-Cepas et al., 2016).

The average nucleotide identity (ANI) values between genomes were estimated by the online web server EzBioCloud (Yoon et al., 2017). tRNA genes were predicted by tRNAscan-SE (Lowe and Chan, 2016), and RNA genes were predicted by RNAmmer1.2 (Lagesen et al., 2007). Secondary metabolite-related genes were predicted by the online web server antiSMASH3.0.5 (Weber et al., 2015). Amino acid composition and protein isoelectric points were counted via the EMBOSS Pepstats website. Signal peptide prediction was performed with SignalP 3.0 (Bendtsen et al., 2004). The MEROPS peptidase database (Release 12.0)^2^ was used to classify the enzymes (Rawlings et al., 2014). A genome circle was constructed using Circos (Krzywinski et al., 2009).

### Comparative Genome Analysis

Clusters of orthologous groups (COGs) of proteins were used for functional classifications performed with the eggNOG (version 4.5) database. Orthologous clusters (OCs) were assigned by grouping all protein sequences from the two genomes using OrthoMCL v2.0.9 software based on their sequence similarity (BLASTP *E*-value less than 1^e–5^, MCL inflation = 1.5) (Fischer et al., 2011). All sequences were first filtered, allowing a minimum protein length of 10 and a maximum stop codon of 20%.

### Morphological, Phenotypic Characteristics and Comparison

#### Morphology

Colonies on marine agar were investigated under a light microscope (SMZ445, Nikon) using cells grown on 2216E agar for 96 h. The individual cells were observed by scanning electron microscopy. The strains were cultured at optimum growth temperature, and when the optical density (OD_600 nm_) reached 0.6, the culture medium was removed. The bacteria were fixed with 2.5% glutaraldehyde for 4 h, washed three times with 0.1M phosphate buffer solution (PBS, pH 7.4), and dehydrated in increasing concentrations of ethanol (30, 50, 70, 90, and 100%) for 20 min each time. Then, the samples were dried in a vacuum freeze dryer. The dried samples were sputter coated with gold and viewed under scanning electron microscopy (Mira 3, Tescan, Czechia).

#### Physiological and Biochemical Characteristics

Hydrolysis of Tween 80, casein, starch, gelatin, and agar was determined on 2216E medium supplemented with 1.0% (v/v) Tween 80, 1% (w/v) casein, 0.5% (w/v) starch, 1% (w/v) gelatin, or 2.0% (v/v) agar, respectively (Nedashkovskaya et al., 2003). Catalase activity was determined by pouring 3% H2O2 solution on bacterial colonies and observing bubble production. Oxidase activities were tested using 1% (w/v) N, N, N9, N9-tetramethyl-*p*-phenylenediamine solutions (Gerhardt, 1994).

#### Optimum Growth Temperature, pH and Salinity Tolerance

*Mesonia algae* K4-1 and *Mesonia* sp. HuA40 cells were cultivated in 2216E medium, and the optimum growth temperature was examined by growing cells at different temperatures (4, 10, 20, 30, 40, and 50°C). The growth effects of pH were measured at pH 4, 5, 6, 7, 8, 9,10, 11, and 12. Culture growth was followed at OD_600 nm_ by Perkin Elmer (United States). The salinity tolerance tests were examined in 2216E liquid medium, *Mesonia algae* K4-1 were cultivated at 18°C and *Mesonia* sp. HuA40 were cultivated at 37°C overnight. When the optical density at 600 nm (OD_600 nm_) reached 0.6, 100 μl of each culture was transferred into 100 ml of 2216E medium containing different concentrations of NaCl (3, 7, 8, 9, 12, 13, 14, and 15%). The OD_600 nm_ value was measured every 8 h on average.

#### Extracellular Protease Activity

The strains were cultured in 500 ml flasks at the optimum growth temperature with shaking at 200 rpm (Wu et al., 2016). The supernatant of the fermentation broth was collected by centrifugation (11,000 × *g*, 4°C, 30 min) after 120 h incubation. The protease activity of the culture supernatant on casein was detected by the Folin phenol method (He et al., 2004). One unit of enzyme activity was determined as the amount of enzyme that catalyzed the formation of 1 μg tyrosine per min.

#### Extracellular Polysaccharide Yield

The fermentation supernatant was added with five volumes of absolute ethanol, mixed, allowed to stand at 4°C for 30 min and centrifuged at 11,000 × *g* for 5 min. Anhydrous ethanol was added to the precipitate, shaken vigorously, and centrifuged at 4°C; the monosaccharide was washed away and repeated three times. The precipitate was dried at 60°C for 30 min and redissolved in distilled water. After the appropriate dilution, the yield of extracellular polysaccharide in the bacterial fermentation broth was determined by the phenol sulfuric acid method (Masuko et al., 2005).

## Results and Discussion

### Classification, Morphology and Characteristics of Strains

**FIGURE 1:**
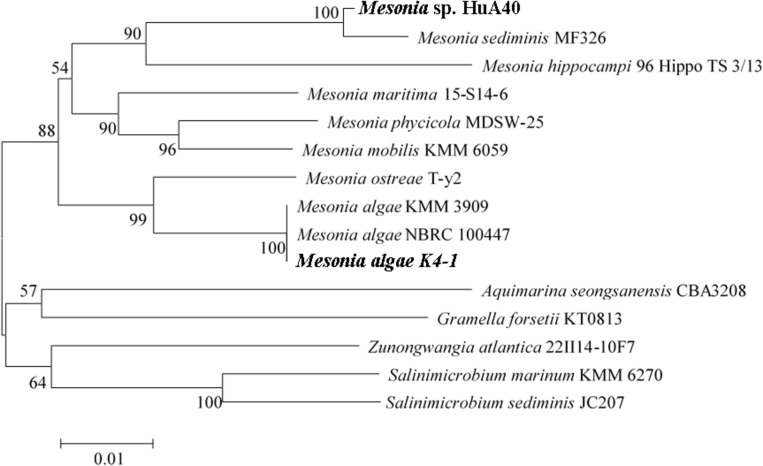
Phylogenetic tree based on the 16S rRNA gene sequences of *Mesonia* sp. HuA40, *Mesonia algae* K4-1 and related strains. The strains *Mesonia* sp. HuA40 and *Mesonia algae* K4-1 are indicated in bold font.

The strain K4-1 was collected in the Arctic ocean (12°07.553E, 78°55.464N), and HuA40 was isolated from hull attachments in Zhanjiang, Guangdong Province, China. The 16S rRNA gene sequences from the genomes of strains K4-1 and HuA40 were subjected to BLAST analysis to identify the closest reference sequences available in GenBank, which showed that the two strains are most closely related to the members of the genus *Mesonia* (Figure 1). The 16S rRNA gene homology between *Mesonia algae* K4-1 and its closest cultured member *Mesonia* algae strain NBRC 100447 is 99.86%. The OrthoANIu value between *Mesonia algae* K4-1 and *Mesonia algae* strain DSM 15361(Accession NZ_QKYV00000000) is 95.28% (>95%). Thus, the two strains are thought to be the same species (Goris et al., 2007). The 16S rRNA gene homology between *Mesonia* sp. HuA40 and its closest cultured member *Mesonia sediminis* MF32f is 99.04%. Because the genomic sequence of *Mesonia sediminis* has not been reported thus far, we defined strain HuA40 from the genus level, *Mesonia* sp. HuA40. In addition, the 16S rRNA gene homology between the two strains was 92%.

**FIGURE 2:**
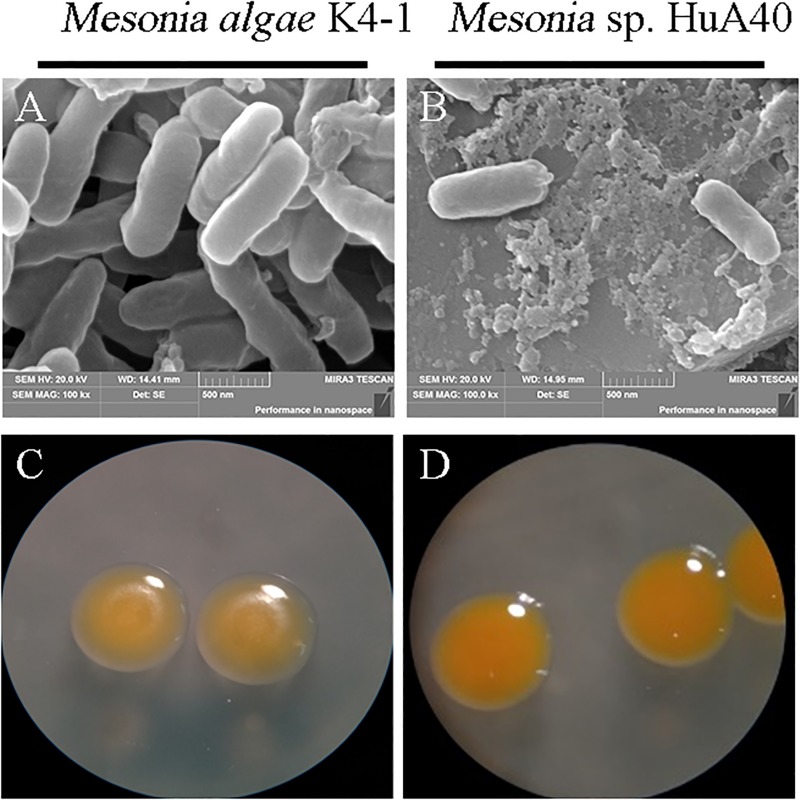
Images of cell morphology. [**(A)** *Mesonia algae* K4-1 and **(B)** *Mesonia* sp. HuA40] Scanning electron microscope (magnification, ×100,000); [**(C)** *Mesonia algae* K4-1 and **(D)** *Mesonia* sp. HuA40] light microscopy pictures in 2216E plate colony (magnification, ×4).

As shown in Figure 2, the morphological characteristics of the two strains were similar. Both strains were rod-shaped and ranged from 0.3 to 0.4 μm in width and from 0.7 to 1.2 μm in length (Figures 2A,B). *Mesonia algae* K4-1 cells were slightly larger than *Mesonia* sp. HuA40 cells. The colonies of these two types were 0.5–1.0 mm in diameter, and circular with entire edges when cultivated on 2216E agar at 16°C for 72 h (Figures 2C,D). Compared with *Mesonia algae* K4-1, the smooth surface of *Mesonia* sp. HuA40 appeared moister and the color was close to orange. On the other hand, the growth curve shows that *Mesonia* sp. HuA40 grew in 3-9% (w/v) NaCl and pH 6.0–8.5, whereas *Mesonia algae* K4-1 grew well even at 3-15% (w/v) NaCl (Table 1). *Mesonia algae* K4-1 could grow at 4°C, and the halo formed by hydrolysis of Tween 80 and the transparent ring formed by the hydrolysis of casein were obviously larger than *Mesonia* sp. HuA40 (Table 1). In addition, *Mesonia algae* K4-1 had a catalase that was missing in *Mesonia* sp. HuA40 (Table 1), which may be related to the tolerance to high concentrations of H_2_O_2_ in the Arctic ocean (Yu et al., 2015). The physiological and biochemical analysis of the two strains showed that the differences were mainly reflected in the tolerance of acid, alkali and salt, growth temperature range, hydrolysis activity of protein and lipids, and hydrogen peroxide decomposition.

**TABLE 1:** Differential phenotypic characteristics of strain *Mesonia algae* K4-1 and *Mesonia* sp. HuA40.

	***Mesonia algae* K4-1**	***Mesonia* sp. HuA40**
Gliding motility	–	–
Colony shape	Round	Round
Colony color	Round	Orange
Oxidase	–	–
Catalase	+	–
**Hydrolysis of:**		
Tween 80	++	+
Casein	++	+
Starch	–	–
Gelatin	+	+
Agar	–	–
Growth at:		
4°C	+	–
5% NaCl	+	+
10% NaCl	+	–
14% NaCl	+	–
**pH for growth:**		
6	+	+
8	+	+
10	+	–

*+Positive, − negative.*

### General Genomic Features

Considering the phenotypic differences between the two strains, we further analyzed the genomic characteristics. General features of the *Mesonia algae* K4-1 and *Mesonia* sp. HuA40 genomes are summarized in Table 2 and Supplementary Figure S1. Overall, draft assemblies of the *Mesonia algae* K4-1 genome yielded 111 contigs with an average GC content of 33.9%. The draft genome is approximately 3.08 Mbp, and a total of 3,190 coding sequences (CDSs) were identified. The *Mesonia* sp. HuA40 genome was automatically assembled into 116 contigs, with an estimated genome size of 2.70 Mbp and a total of 2585 CDSs. The Arctic bacterium *Mesonia algae* K4-1 has a larger genome and a higher proportion of CDSs than the *Mesonia* sp. HuA40 genome. Bentkowski et al. (2015) established a correlation model to study the genome size evolution of populations at different environmental disturbance levels. It is proposed that different environmental variations lead to more genes in the genome. Konstantinidis and Tiedje (2004) proposed that strains with relatively large genomes could buffer environmental disturbances more effectively. The genome analysis results showed that *Mesonia algae* K4-1 may be more robust to variable conditions and equipped with an ecological advantage. Horizontal gene transfer (HGT) is a general way for bacteria to respond to different selective pressures and obtain an important competitive advantage (Chen et al., 2016). In HGT events, the acquisition or loss of MGE plays a key role in carrying functional genes that enhance fitness in the environment (Hall et al., 2017; Wang L. et al., 2017; Husnik and McCutcheon, 2018). In general, the number of transposable elements is relatively high in large genomes, indicating that gene turnover is relatively frequent and has metabolic diversity (Gweon et al., 2017). As shown in Table 3, 11 transposase- and 13 integrase-encoding genes were found in the genome of strain *Mesonia algae* K4-1, while *Mesonia* sp. HuA40 encodes 8 transposases and 3 integrases. The increased number of copies of the enzyme support that *Mesonia algae* K4-1 has a larger genome than *Mesonia* sp. HuA40. In addition to potential HGT activities, these adaptive genes move between different species, promoting the diversity of bacteria in different environments. *Mesonia algae* K4-1 and *Mesonia* sp. HuA40 encode 78 and 51 transcriptional regulators, respectively, suggesting that *Mesonia algae* K4-1 may be more adaptable in a wider range of external and gradient-affected environments.

**TABLE 2:** Comparison of genome features of *Mesonia algae* K4-1 and *Mesonia* sp. HuA40.

**Attributes**	***Mesonia algae* K4-1**	***Mesonia* sp. HuA40**
Total Length (bp)	3435282	2701467
G + C content	33.9%	36.09%
Contig number	111	27
Contigs (≥1000 bp)	70	85
Average length (bp)	30947.49	21271.39
N50 contig length (bp)	182132	207604
Number of tRNAs	41	39
Number of genes	3190	2585
Number of RNAs	43	42

**TABLE 3:** Comparison of the numbers of selected proteins between *Mesonia algae* K4-1 and *Mesonia* sp. HuA40.

**Category**	**Description**	***Mesonia* sp. HuA40**	***Mesonia algae* K4-1**
(1) Enzymes for degradation	Chitinase	0	2
	Esterase	29	35
	Peptidase	167	223
(2) Polysaccharide biosynthesis	Glycosyltransferase	41	54
(3) Oxidoreductases	D-lactate dehydrogenase	1	2
	Alcohol dehydrogenase	1	7
	Aldehyde dehydrogenase	2	5
(4) Salt and cold adaptation	Na^+^/H^+^ antiporter protein	11	12
	Glycine betaine transporter OpuD	0	1
	Glycine/sarcosine N- methyltransferase	0	1
	Helicases	14	23
Other genes			
(5) Transcriptional regulator		51	78
(6) Integrase		3	13
(7) Transposase		8	12

### Differential Gene Content

**FIGURE 3:**
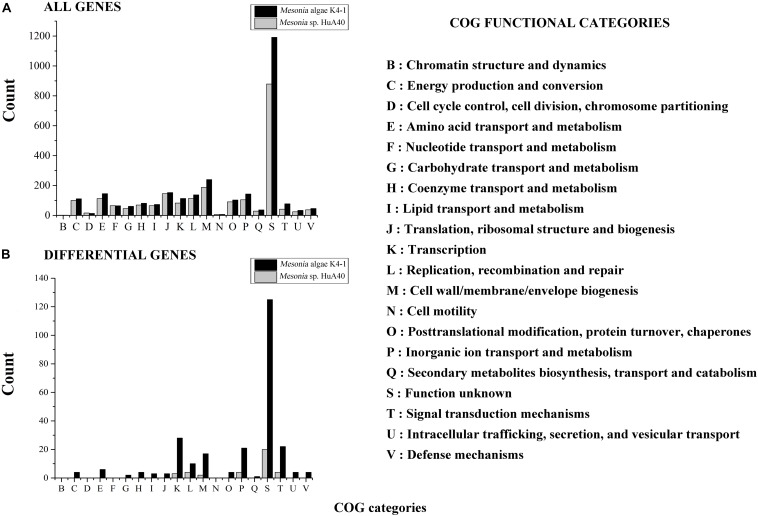
Distribution of COG functional classes. Count of COGs predicted in *Mesonia algae* K4-1 and *Mesonia* sp. HuA40 genomes. All genes **(A)** and genes found only in one of the genomes **(B)** are indicated. COG, clusters of orthologous groups.

All genes of *Mesonia algae* K4-1 and *Mesonia* sp. HuA40 were functionally classified according to the COG category. In total, 88.04% of *Mesonia* sp. HuA40 genes and 88.53% of *Mesonia algae* K4-1 genes were annotated with the COG database (Figure 3A). To evaluate the degree of genetic differences encoded by the two strains, the unique genes of each strain were classified by COG categories, and the frequencies of the two genomes were compared (Figure 3B and Supplementary Table S1). The results showed that *Mesonia algae* K4-1 has a higher number of unique genes in COG K (transcription), COG T (signal transduction mechanism), COG P (inorganic metal ion transport and metabolism), COG L (replication, recombination and repair), and COG M (cell wall, membrane and envelope biogenesis). The increase in COG K, COG T revealed that *Mesonia algae* K4-1 may possess a more complex transcriptional system and strong regulatory systems to respond to various environmental stimuli and change the transcription rate by altering the level of gene expression. Moreover, the grouping of COG M shows that *Mesonia algae* K4-1 encodes specific genes related to polysaccharide synthesis, outer membrane proteins, and lipid synthesis. The unique genes of COG P encoded by *Mesonia algae* K4-1 may suggest mechanisms that maintain the specificity of osmotic adjustment. Interestingly, genomic analysis showed that the strain *Mesonia algae* K4-1 encodes approximately 42.17% of the proteins belonging to the unknown functional COG, which contains 1,191 proteins. In contrast, *Mesonia* sp. HuA40 encodes 39.8% proteins of unknown function, which contains 879 proteins. This result indicated that the *Mesonia algae* K4-1 currently has large number of proteins of unknown functions.

### Salinity Adaptation Strategy and pH Regulation

**FIGURE 4:**
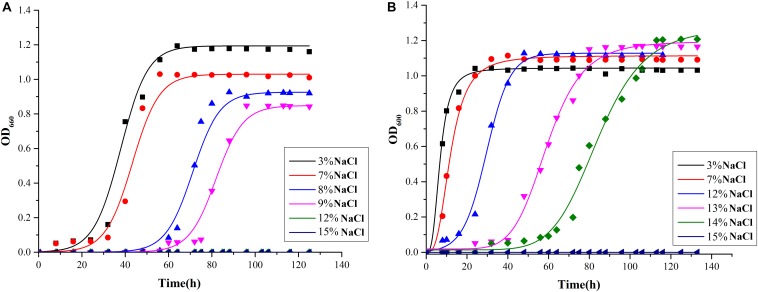
Growth curves of *Mesonia* sp. HuA40 **(A)** and *Mesonia algae* K4-1 **(B)** in different NaCl concentrations.

As shown in Figure 4, *Mesonia algae* K4-1 tolerated 3–14% NaCl, while strain *Mesonia* sp. HuA40 only tolerated 9% NaCl. At 3% and 7% NaCl, the lag phase of *Mesonia algae* K4-1 was shorter than that of *Mesonia* sp. HuA40. Moreover, *Mesonia algae* K4-1 had a much higher cell density at stationary phase when the NaCl concentration was increased to 14%. The *Mesonia* sp. HuA40 biomass gradually decreased in stationary phase when the NaCl concentration was increased to 9%. These results indicated that the salt tolerance of *Mesonia* sp. HuA40 was significantly lower than that of *Mesonia algae* K4-1. Due to the high salinity of seawater accompanied by high pH (Collins et al., 2010), we also tested the pH tolerance of the two strains. *Mesonia algae* K4-1 can tolerate pH 6–10, while *Mesonia* sp. HuA40 can only tolerate pH 6-8 (Supplementary Figure S2). This result is consistent with the expectation that salt-tolerant bacteria have strong alkali tolerance (Oren, 1999).

The moderate halophilic bacterium *Mesonia algae* K4-1 must have a highly efficient, salt-tolerance mechanism unique to *Mesonia* sp. HuA40. To further clarify the salt tolerance mechanism of *Mesonia algae* K4-1, we further analyzed and compared the adaptation mechanism of *Mesonia algae* K4-1 and *Mesonia* sp. HuA40 to the marine salt environment. Usually, halophilic microorganisms have two strategies for maintaining suitable osmotic pressure in the cytoplasm (Oren, 2008; Joghee and Jayaraman, 2016). The first strategy involves accumulation of inorganic ions (K+/Na+) of equal molar concentration in cells (Oren, 2013a). The intracellular proteins of bacteria relying on this strategy to maintain structural stability and physiological activity at high salt concentrations and exhibit remarkable instability at low salt concentrations (Lentzen and Schwarz, 2006). The proteomes of such bacteria exhibit a high degree of acidity, and microorganisms that normally rely on this strategy are obligate halophiles, such as *Archaea* (Siglioccolo et al., 2011). The second strategy is to synthesize or accumulate organic solutes such as secretin, glycine betaine, trehalose, amino acids and derivatives, which are osmotic substances that maintain the intracellular and extracellular osmotic balance and reduce the accumulation of intracellular salt (Welsh, 2000; Saum et al., 2013). *Mesonia algae* K4-1 has the glycine betaine transporter OpuD and the choline/glycine/valine betaine transporter BetT (driven by proton or sodium motive force, can also transport betaine or choline and, in some instances, proline), both of which are not annotated in *Mesonia* sp. HuA40. Glycine betaine is small in size and relatively low in energy (Gunasekera et al., 2008). Although glycine betaine is a relatively favorable organic solute, only a few archaea that live in a highly permeable environment can synthesize it. Additionally, one of the two methyltransferases that catalyze the glycine to betaine three-step reaction, encoded by the gsmT gene, was only found in the *Mesonia algae* K4-1 genome. Moreover, glycine/sarcosine *N*-methyltransferase (GSMT), a key protein for the biosynthesis of glycine betaine from glycine, allows the accumulation of sarcosine and is considered an osmotic protector (Nyyssola et al., 2000; Raiger Iustman et al., 2015). Secondary transporters of the betaine/choline/carnitine transporter family (BCCT) can also transport betaine or choline and, in some instances, proline (Ziegler et al., 2010). This indicated that *Mesonia algae* K4-1 can use an organic compatible solutes strategy to maintain its cell osmotic balance in the face of high osmotic pressure.

Bacteria that generally use this strategy require only a small amount of protein to adapt and have a wide range of salt ion tolerance (Roberts, 2005; Oren, 2013b). Therefore, we further compared the characteristics of intracellular and extracellular proteins between *Mesonia algae* K4-1 and *Mesonia* sp. HuA40. In *Mesonia algae* K4-1, the extracellular proteins with signal peptides had a higher ratio of acidic residues to basic residues than the intracellular proteins (Table 4). The predicted isoelectric point (pI) of the extracellular proteins with signal peptides was lower (*p* < 0.05) than that of the intracellular proteins without signal peptides (*p* < 0.05). *Salinibacter ruber*, which has been studied as an extreme halophilic bacterium, maintains cell osmotic balance by increasing the intracellular ion concentration in high salt environments (Oren, 2013a; Sanjukta et al., 2013). Both intracellular and extracellular proteins of *S. ruber* are tolerant to high salt environments, and the intracellular and extracellular pI was significantly lower than that of non-halophilic *Escherichia coli* (Qin et al., 2010). The salt-tolerance of the extracellular proteins of strain K4-1 and the genome annotation results indicated that *Mesonia algae* K4-1 can maintain its cell osmotic balance by using glycine betaine and proline, glutamate and other uptake systems. This strategy was also applied by the moderate halophilic strain *Zunongwangia profunda* SM-A87, which also contains a glycine betaine transporter (Qin et al., 2010). In addition, the pI of both intracellular and extracellular proteins of *Mesonia algae* K4-1 was lower than that of *Mesonia* sp. HuA40; the protein of the halophilic bacterium *Mesonia algae* K4-1 may have a certain preadaptation ability to a high salt environment. As previously demonstrated, the basic mechanism of salinity adaptation of *Mesonia algae* K4-1 is to prevent a large amount of inorganic salts from entering the cells and to use organic permeate to balance the high salinity of the environment. Further research at the transcriptional and translational levels is needed to elucidate the exact mechanism of salinity adaptation.

**TABLE 4:** Properties of intracellular and extracellular proteins of *Mesonia algae* K4-1 and *Mesonia* sp. HuA40.

	***Mesonia algae* K4-1 All proteins**		***Mesonia* sp. HuA40 All proteins**	
		
	**With Signal peptides**	**Without signal peptides**	**With signal peptides**	**Without signal Peptides**
Number	355	2794	280	2188
Asp (percentage)	6.15	5.28	5.75	5.09
Glu (percentage)	6.36	7.30	5.91	6.82
Arg (percentage)	2.88	3.39	3.19	3.63
Lys (percentage)	6.37	8.27	6.51	7.30
(Asp + Glu)/(Lys + Arg)	1.35	1.07	1.24	1.00
pI	6.00 ± 2.09	7.21 ± 2.16	6.47 ± 2.23	7.66 ± 2.13

*pI, predicted isoelectric point. The data are averages with standard deviation.*

Since the high salt environment is generally accompanied by a high alkali environment (Padan et al., 2005), to study the tolerance of the two strains to pH, we also measured the growth of the two strains under different pH conditions. The urease, arginine deaminase and arginine dehydrogenase systems (Ads), which help bacteria survive in acidic conditions by producing alkaline substances, were missing in *Mesonia algae* K4-1 and *Mesonia* sp. HuA40. This result may be related to the weak alkaline character of seawater (Liu et al., 2015). Intriguingly, *Mesonia algae* K4-1 has 12 genes encoding Na^+^/H^+^ antiporter proteins, including two genes encoding NhaB type Na^+^/H^+^ antiporter proteins, a gene encoding an NhaC type Na^+^/H^+^ antiporter protein, and three NhaP type Na^+^/H^+^ antiporter proteins. These antiporters play a crucial part in maintaining pH homeostasis and empowering salt tolerance (Herz et al., 2003; Klotz et al., 2006). Expect the single subunit Na^+^/H^+^ antiporter, *Mesonia algae* K4-1 contains the multisubunit Mrp-like Na^+^/H^+^ antiporter. As a hetero-oligomer, the Mrp system, broadly exists in the phylogenetic process of bacteria and archaea. The physiological effects of the Mrp system have been confirmed in alkaline tolerance, Na^+^ tolerance and K^+^ tolerance. Compared with *Mesonia algae* K4-1, *Mesonia* sp. HuA40 also encodes the Mrp-like Na^+^/H^+^ antiporter. The difference is that in addition to a gene encoding an NhaC type Na^+^/H^+^ reverse transport, *Mesonia* sp. HuA40 possesses a gene encoding an NhaD type Na^+^/H^+^ antiporter. This NhaD type Na^+^/H^+^ antiporter was nearly identical to that of the moderate halophilic bacterium *Psychroflexus salaries* and presented 82.4% identity and 95% similarity with the NhaD gene. Almost all halophilic microorganisms have the ability to expel Na^+^ from the interior of cells and maintain cytoplasmic pH homeostasis under alkaline conditions using Na^+^/H^+^ antiporters (Wang Y. et al., 2017). Analysis of these genes helps to understand the regulation of ion concentration and pH homeostasis under environmental change.

### Cold Adaptation

Cold-adapted bacteria can be successfully grown under extreme conditions of cold marine environments because of various structural and physiological adjustments in the genome (Russo et al., 2010). *Mesonia algae* K4-1 was isolated from the Arctic, and our study found that it can thrive at 4°C (data not shown). However, *Mesonia* sp. HuA40 cannot grow at low temperature. To better understand the cold-adapted mechanism of the strain, we used genomic data to study and compare the traits of two strains, *Mesonia algae* K4-1 and *Mesonia* sp. HuA40, for cold adaptation (Table 3). When temperatures drop, bacteria must sense environmental changes and adjust their metabolism. Histidine kinases act as a multifunctional sensory to control numerous cold-responsive genes as well as many different stimuli (Sinetova and Los, 2016; Tang et al., 2019). However, in the *Mesonia algae* K4-1 genome, 30 genes encoding histidine kinases were found. Only 15 histidine kinases were encoded in the *Mesonia* sp. HuA40 genome. This result indicated that *Mesonia algae* K4-1 has a more powerful ability to respond to external stimuli. Glutathione synthase (gshB), a key enzyme for glutathione synthesis that has a key function in maintaining cell redox homeostasis, osmotic stress and protecting membrane lipids from the oxidative stress induced at cold temperatures (Loi et al., 2015; Mocali et al., 2017), was only encoded in the *Mesonia algae* K4-1 genome. This result suggests that *Mesonia algae* K4-1 faces high ROS concentrations, and glutathione likely helps *Mesonia algae* K4-1 to cope with oxidative stress in cold environments. When bacteria are exposed to cold stimulation, their growth will stagnate for a short time. This is because low temperature leads to the formation of more stable secondary structures in the process of translation, which inhibits initiation and extension. However, bacteria overcome growth stagnation by cold-induced RNA helicase, which contributes to the formation of cold-adapted ribosomes and RNA degradosomes, implying a role in unwinding the RNA secondary structure stabilization at low temperature (Owttrim, 2006, 2013). A total of 8 and 3 RNA helicases were annotated in *Mesonia algae* K4-1 and *Mesonia* sp. HuA40, respectively, suggesting that RNA helicase may play a role in the normal cellular function of *Mesonia algae* K4-1 at lower than optimal growth temperature. Furthermore, the organic compatible solutes of *Mesonia algae* K4-1 also encoded cryoprotection to maintain its cell normal physiological function at low temperatures (Wemekamp-Kamphuis et al., 2004). Some studies have reported that psychrophilic proteins are generally characterized by a higher degree of structural flexibility, lower thermostability, and higher specific activity (De Maayer et al., 2014). Therefore, we analyzed the amino acid composition ratios of different side chain sizes in *Mesonia algae* K4-1 and *Mesonia* sp. HuA40 proteins (Table 5). The amino acid composition of *Mesonia algae* K4-1 protein is characterized by more small residues such as Gly, Ser, Thr, and Asn and less large side chain amino acids, such as Arg, Leu, and Lys. The results are consistent with the amino acid composition of the cold-adapted proteins that are characterized by more small residues such as Gly and non-charged polar amino acids, particularly Gln and Thr, and less hydrophobic amino acids, particularly Leu, compared with their mesophilic counterparts (Yang et al., 2015).

**TABLE 5:** Comparison of the side chain size between *Mesonia algae* K4-1 and *Mesonia* sp. HuA40 protein.

**Amino acids**	**Percentage**	
	
	***Mesonia algae* K4-1**	***Mesonia* sp. HuA40**
**Small side chain**		
Gly	6.11	6.02
Ser	6.59	6.04
Thr	5.62	5.27
Asn	6.23	6.07
(Gly + Ser + Thr + Asn)	24.56	23.40
**Large side chain**		
Arg	3.32	3.56
Leu	9.46	9.99
Lys	8.00	8.04
(Arg + Leu + Lys)	20.78	21.58

**FIGURE 5:**
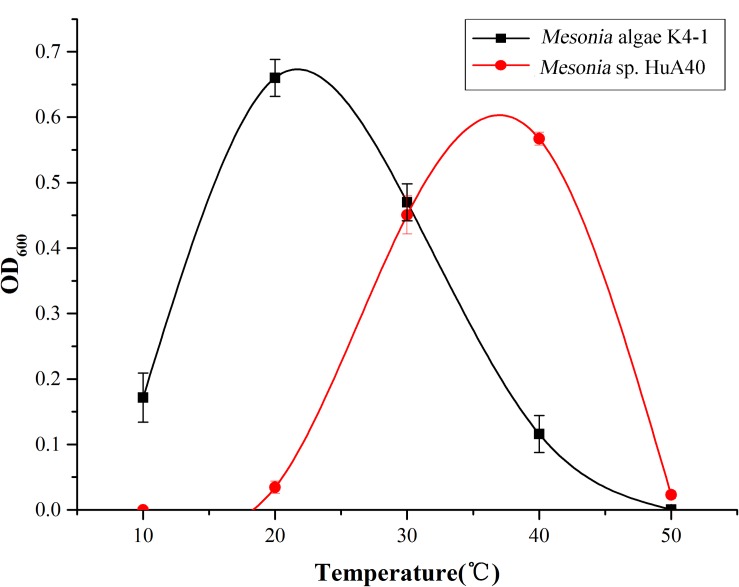
Optimal growth temperature of the *Mesonia* sp. HuA40 and *Mesonia algae* K4-1.

The growth curve shows that the optimum growth temperature of *Mesonia algae* K4-1 is close to 20°C, while that of *Mesonia* sp. HuA40 is close to 40°C (Figure 5). The growth temperature range of *Mesonia algae* K4-1 of 10–50°C is obviously wider than that of *Mesonia* sp. HuA40, which is 20–50°C. Moreover, *Mesonia algae* K4-1 can grow at a low temperature of 4°C, while *Mesonia* sp. HuA40 growth is stagnant at low temperature. These results suggested that *Mesonia algae* K4-1 has a stronger cold adaptation ability.

### Polysaccharide Biosynthesis

**FIGURE 6:**
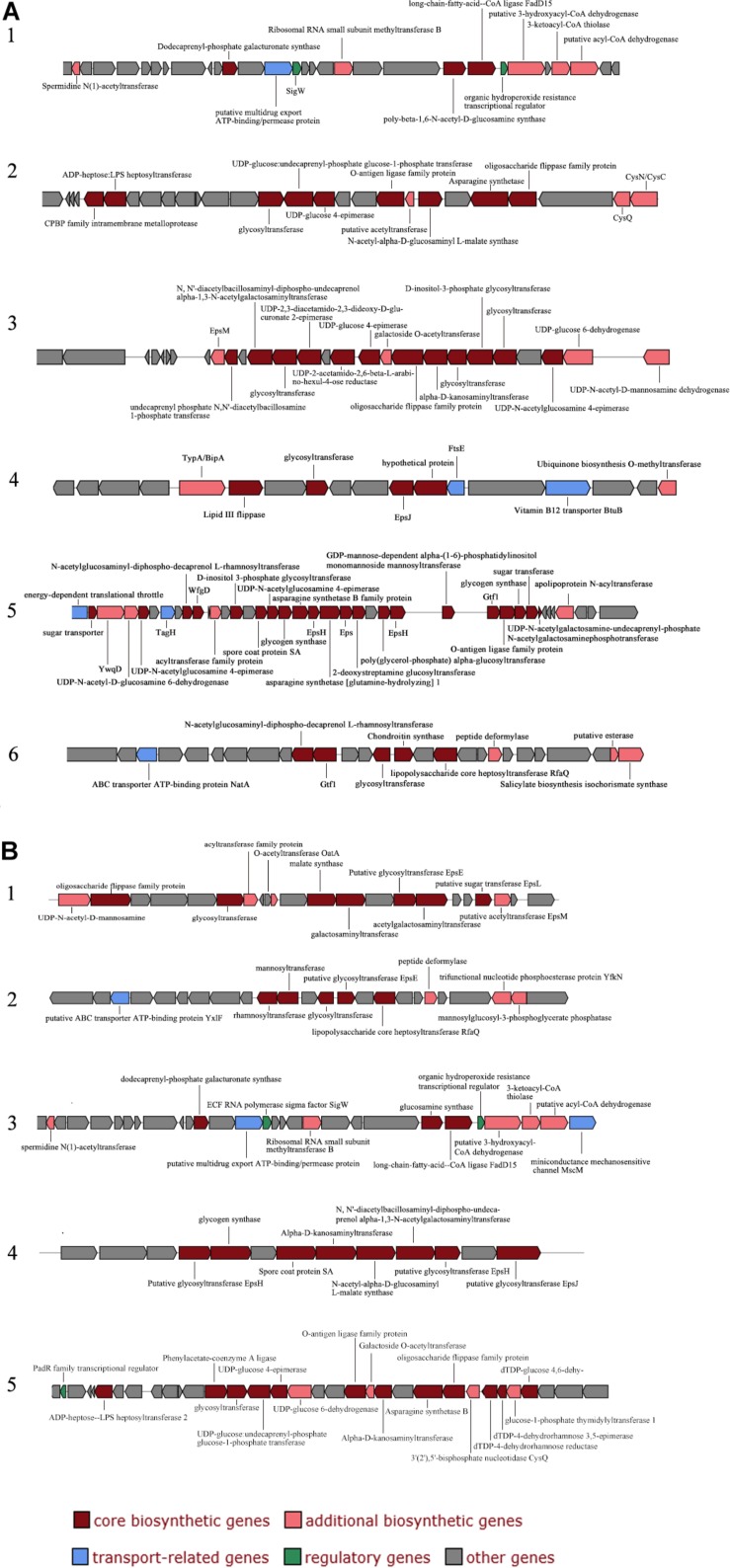
Organization of saccharide cluster structural genes in *Mesonia algae* K4-1 **(A)** and *Mesonia* sp. HuA40 genomes **(B)**.

The extracellular polysaccharides produced by many marine bacteria give them the ability to survive in extreme marine environments, helping bacteria concentrate organic matter, absorb metal ions (Zhang et al., 2017), form biofilms, and tolerate high acidity, UV radiation (Nicolaus et al., 2010), low temperatures and high salinities (Nichols et al., 2005; Kazak et al., 2010). To study the ecological role of *Mesonia algae* K4-1 exopolysaccharide in cold Arctic environments, the saccharide biosynthetic gene clusters were compared and analyzed (Figure 6). Using antiSMASH3.0.5 to predict secondary metabolite biosynthesis gene clusters, we found that the *Mesonia algae* K4-1 genome has 6 genes clusters related to saccharide biosynthesis, whereas *Mesonia* sp. HuA40 contains 5. Glycosyltransferase is the most important enzyme in the synthesis of polysaccharides (Cantarel et al., 2009; Liang et al., 2015). The number of glycosyltransferase types determines the number of monosaccharides in polysaccharide repeat units and the types of glycosidic bonds connected to each other. The genome annotation revealed that *Mesonia algae* K4-1 harbors 54 predicted glycosyl transferases, of which 12 belong to family two and 22 belong to family one. Among the 41 glycosyltransferase genes encoded by *Mesonia* sp. HuA40, 17 belong to the glycosyltransferase I family, and 10 belong to the glycosyltransferase II family. The discovery of polysaccharide exporter membrane proteins Wza, EpsA, etc., indicated that *Mesonia algae* K4-1 and *Mesonia* sp. HuA40 had the ability to secrete extracellular polysaccharides. After grown in fermentation culture for 4 days, the maximum EPS production of *Mesonia algae* K4-1 reached 9.86 mg/ml, while *Mesonia* sp. HuA40 was able to produce 7.96 mg/ml (Supplementary Figure S3). Our experimental results show that *Mesonia algae* K4-1 had higher extracellular polysaccharide production than *Mesonia* sp. HuA40. The extracellular polysaccharides of the Arctic sea ice bacterium *Pseudoalteromonas* sp. SM20310 could significantly enhance the high-salinity tolerance of SM20310 and improve the survival of SM20310 after freeze-thaw cycles, which enables the strain to adapt to a low temperature, high salinity sea ice environment (Liu et al., 2013). The large quantities of extracellular polysaccharides are beneficial to *Mesonia algae* K4-1 for growth in the Arctic ocean.

### Proteolytic Capacity

**FIGURE 7:**
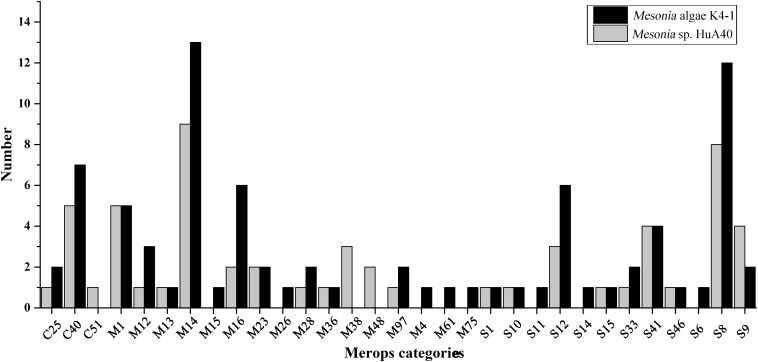
The MEROPS category of the extracellular peptidases from *Mesonia algae* K4-1 and *Mesonia* sp. HuA40.

Bacterial proteases are widespread enzymes that play important roles in cell viability, stress response and so on (Culp and Wright, 2017). They can catalyze the conversion of high molecular weight organics into small substrates to be transported into cells for further processing (Ran et al., 2014). To compare the hydrolysis ability of the extracellular proteins of the two strains, the two strains were subjected to fermentation culture, and the enzyme activity of the extracellular protein was measured (Supplementary Figure S4). Both strains reached maximum enzyme activity on the fourth day of fermentation, while the extracellular enzyme activity of *Mesonia algae* K4-1 was significantly higher than that of *Mesonia* sp. HuA40. *Mesonia algae* K4-1 contains 223 peptidases and *Mesonia* sp. HuA40 has 167. The extracellular peptidases encoded by the two strains were classified using the MEROPS peptidase database; the extracellular peptidases belong mainly to the family of metal peptidases and serine peptidases, as shown in Figure 7. All the extracellular peptidases of *Mesonia algae* K4-1 were classified into 22 different families, and 41 *Mesonia* sp. HuA40 peptidases with signal peptides could be assigned to 17 different families. Most of the enzymes of *Mesonia algae* K4-1 belongs to the M1 and S8 family. The diversity of the extracellular peptidase family and the high activity of extracellular proteases indicated that *Mesonia algae* K4-1 has a higher ability to degrade proteinaceous organic nitrogen and to acquire nutrients from the complex environment.

## Conclusion

This work presents the first complete genome of the genus *Mesonia*. The genome of the polar ocean bacterium *Mesonia algae* K4-1 was described and compared with the tropical sea-water bacterium *Mesonia* sp. HuA40. Our study found that *Mesonia algae* K4-1 had a larger genome and a higher proportion of genes involved in transcription and signal transduction, indicating that *Mesonia algae* K4-1 might have a more powerful ability to respond to various environmental stimuli. Strain *Mesonia algae* K4-1 can tolerate 3–14% NaCl and high pH conditions, while *Mesonia* sp. HuA40 can only grow under 3–9% NaCl and pH 6–8. Comparative genomic analysis revealed the salt tolerance mechanism of the moderately halophilic bacterium *Mesonia algae* K4-1 by synthesizing or accumulating organic solutes to maintain osmotic balance under high osmotic pressure. In addition, *Mesonia algae* K4-1 thrived at low temperature because *Mesonia algae* K4-1 harbored a variety of genes related to cold adaptation, such as RNA helicase, glutathione, and organic compatible solutes. In addition, we found that *Mesonia algae* K4-1 synthesized a large number of extracellular polysaccharides and secreted various families of extracellular proteases to degrade organic nitrogen, which might be related to the adaptation of *Mesonia algae* K4-1 to extremely oligotrophic environments. Our findings provide new insights into the genomic features of *Mesonia* and the adaptive characteristics of different microorganisms in extreme environments.

## Data Availability Statement

This Whole Genome Shotgun project has been deposited at DDBJ/ENA/GenBank under the accession VRLS00000000 (*Mesonia algae* K4-1) and VRLT00000000 (*Mesonia* sp. HuA40). The version described in this article is XXXX01000000. The genomic data for this study are available at https://www.ncbi.nlm.nih.gov/Traces/wgs/VRLS01?display=contigs, https://www.ncbi.nlm.nih.gov/Traces/wgs/VRLT01?display=contigs.

## Author Contributions

RH performed the experiments and prepared the manuscript draft. HH, CY, and DX conceived and designed the experiments. HH and JH helped to revise the manuscript. DL and MW assisted in the formatting of the figures. CL and YZ assisted in data analysis. All authors read and agreed to the final content.

## Conflict of Interest

The authors declare that the research was conducted in the absence of any commercial or financial relationships that could be construed as a potential conflict of interest.

## Footnotes

1 http://www.vicbioinformatics.com/software.prokka.shtml

2 http://merops.sanger.ac.uk
