# Supplementary material for: Genome Sequencing of Mesonia algae K4-1 Reveals Its Adaptation to the Arctic Ocean
Source: Front Microbiol. 2019 Dec 4;10:2812. doi: 10.3389/fmicb.2019.02812 (PMC6905171; doi:10.3389/fmicb.2019.02812)
Supplement: Supplementary file 1 [file Table_1.docx]

**
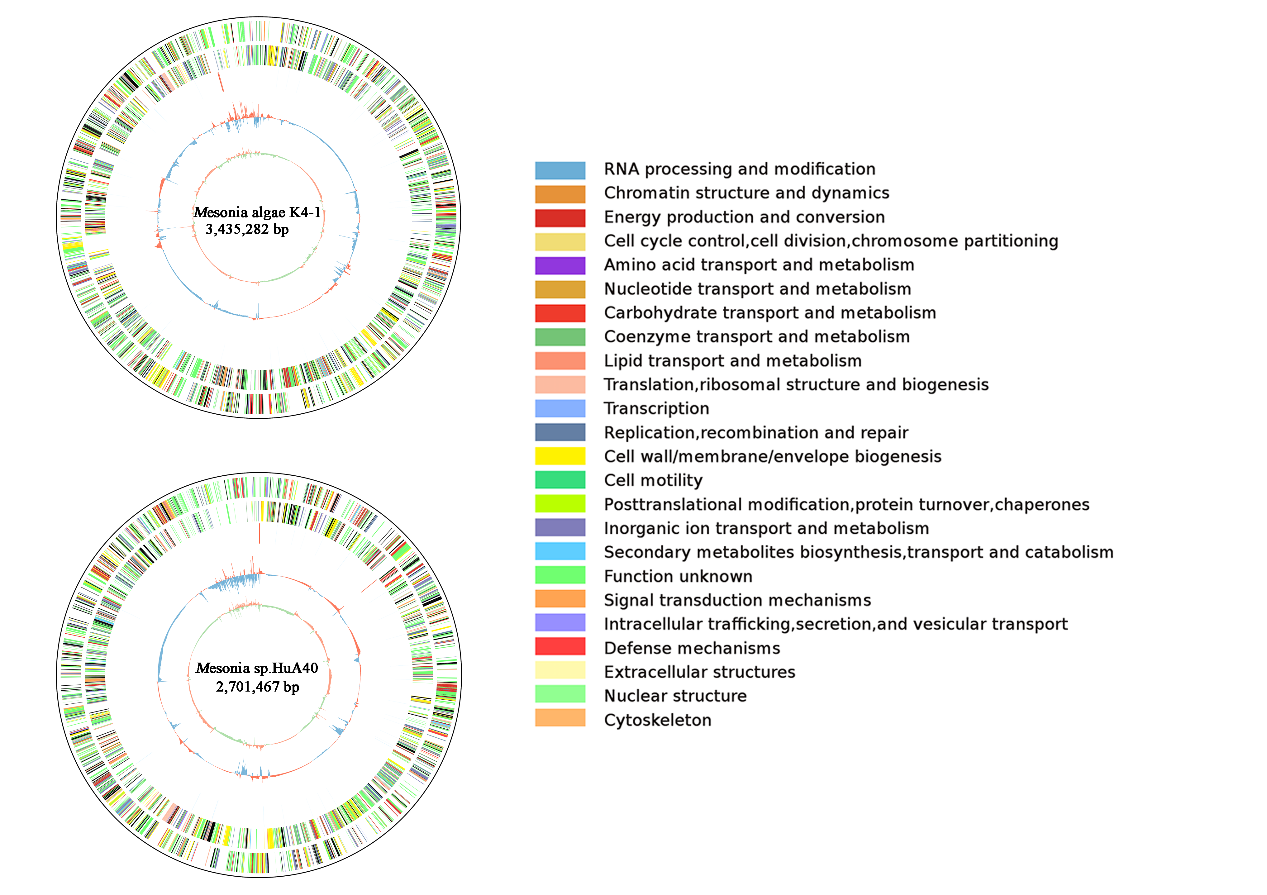
Figure S1. Circular representation of the *Mesonia algae* K4-1 and *Mesonia* sp. HuA40 genome.** Circular chromosome of *Mesonia algae* K4-1 and *Mesonia* sp. HuA40.The scale on the outside indicates the size. Rings 1 and 2 (from the outside in) indicate the genes in forward and reverse strands respectively, the colors of the genes indicate the COG categories. Rings 4 represents rRNA and tRNA. Rings 5 and 6 indicate the G+C content and GC skew [(C+G)/(C+G)], respectively. Circular genome map was generated by Circos.


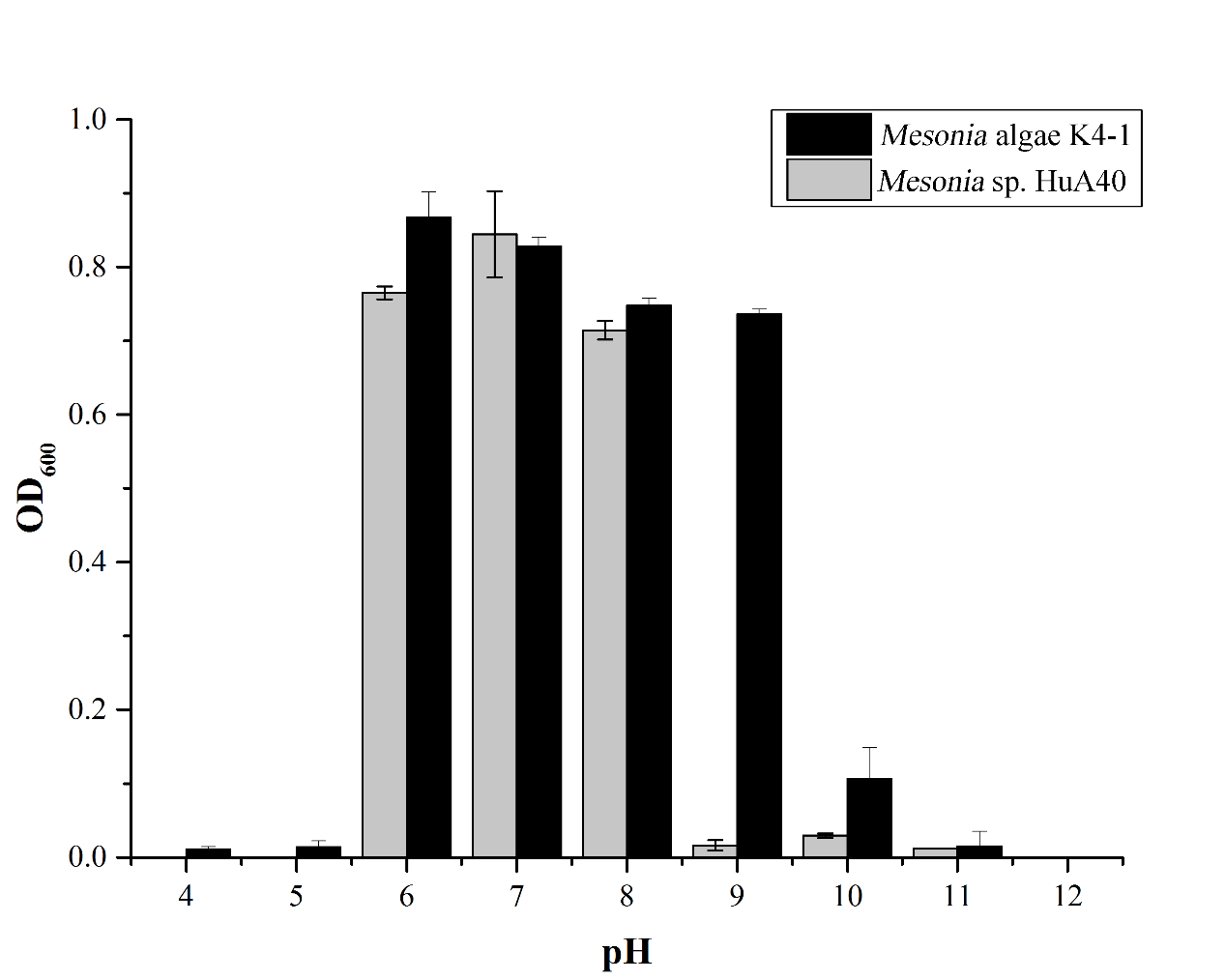


**Figure S2. pH tolerance of the *Mesonia* sp. HuA40 and *Mesonia algae* K4-1.**

**
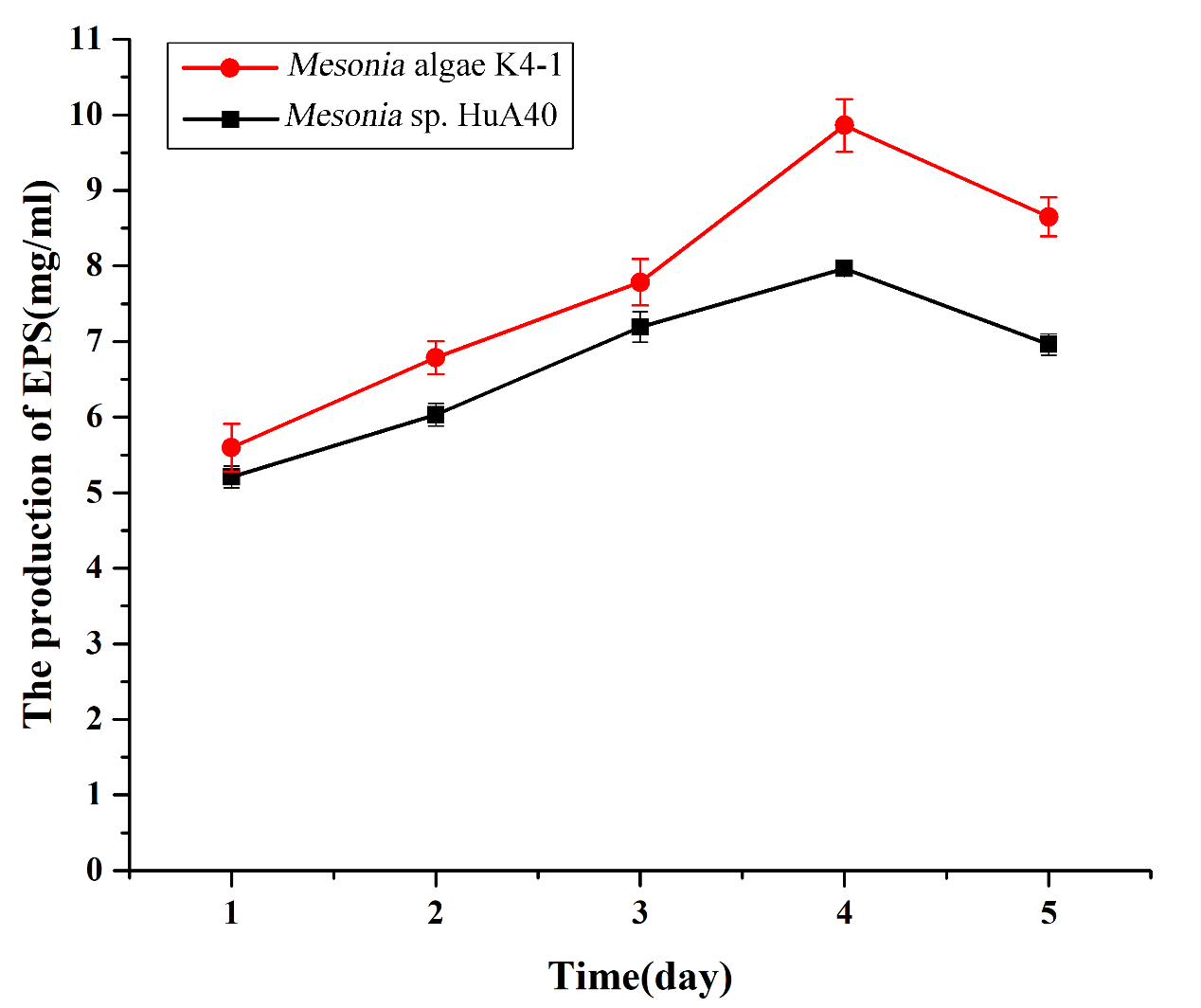
Figure S3. The extracellular** **polysaccharide yield by *Mesonia algae* K4-1 and *Mesonia* sp. HuA40 at different fermentation times**

**
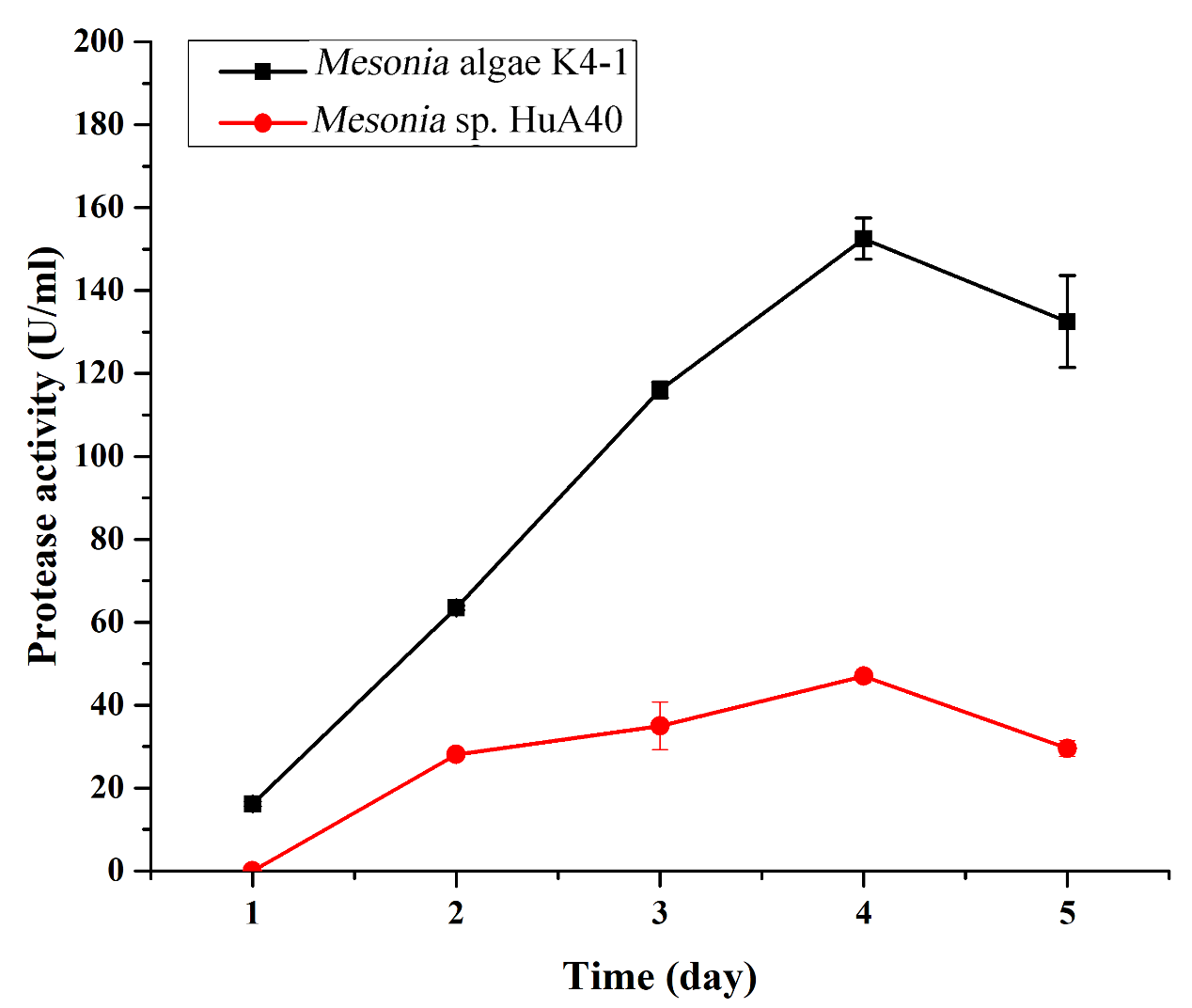
Figure S4. Activity of extracellular protease produced by *Mesonia algae* K4-1 and *Mesonia* sp. HuA40 at different fermentation times.**

**Table S1. Number of genes associated with general COG functional categories in the two *Mesonia* strains**

| COG  category | Description | *Mesonia* sp. HuA40 count | *Mesonia algae* K4-1 count |
| --- | --- | --- | --- |
| B | Chromatin structure and dynamics | 1 | 1 |
| C | Energy production and conversion | 100 | 111 |
| D | Cell cycle control, cell division, chromosome partitioning | 16 | 13 |
| E | Amino acid transport and metabolism | 112 | 145 |
| F | Nucleotide transport and metabolism | 65 | 64 |
| G | Carbohydrate transport and metabolism | 45 | 60 |
| H | Coenzyme transport and metabolism | 69 | 81 |
| I | Lipid transport and metabolism | 65 | 73 |
| J | Translation, ribosomal structure and biogenesis | 145 | 152 |
| K | Transcription | 82 | 113 |
| L | Replication, recombination and repair | 112 | 137 |
| M | Cell wall/membrane/envelope biogenesis | 187 | 239 |
| N | Cell motility | 6 | 6 |
| O | Posttranslational modification, protein turnover, chaperones | 90 | 103 |
| P | Inorganic ion transport and metabolism | 104 | 143 |
| Q | Secondary metabolites biosynthesis, transport and catabolism | 28 | 36 |
| S | Function unknown | 879 | 1191 |
| T | Signal transduction mechanisms | 41 | 77 |
| U | Intracellular trafficking, secretion, and vesicular transport | 24 | 33 |
| V | Defense mechanisms | 37 | 46 |
| Total number | - | 2208 | 2824 |
